# BCR‐ABL exon 7 deletion and novel point mutation in patient with chronic myelogenous leukemia

**DOI:** 10.1002/ccr3.2118

**Published:** 2019-05-10

## AUTHORSHIP LIST

1. Adhamjon Abdullaev, MD, PhD, Senior Research Scientist

Molecular Hematology Department, National Research Center for Hematology, Moscow, Russia.

*Authorship*: Designed and performed sequencing experiments, analyzed the data, and interpreted the results.

Email: adham_abdullaev@mail.ru

2. Irina Nemchenko, MD, PhD, Senior Research Scientist

Department of Chemotherapy of Myeloproliferative disorders, National Research Center for Hematology, Moscow, Russia.

*Authorship*: Involved in patient management and follow‐up, analysis of clinical data.

Email: isn1965@mail.ru

3. Olga Nesterova, Undergraduate Student, Faculty of Fundamental Medicine, Lomonosov Moscow State University

*Authorship*: Interpreted the results.

Email: oy.nesterova@gmail.com

4. Ilya Mikhailov, Undergraduate Student, Faculty of Fundamental Medicine, Lomonosov Moscow State University

*Authorship*: Wrote and submitted the manuscript.

Email: ilyamikhailov5@gmail.com

5. Andrey Sudarikov, PhD, DSc, Head of Molecular Hematology Department

*Authorship*: Contributed to the design of the research and data analysis.

Email: dusha@blood.ru

Mikhailov I. (2018) BCR‐ABL exon 7 deletion and novel point mutation in patient with chronic myelogenous leukemia and TKI resistance. Clin Case Rep, 6: 2057‐2060. https://doi.org/10.1002/ccr3.1794

